# Synthesis of Porous Biochar Containing Graphitic Carbon Derived From Lignin Content of Forestry Biomass and Its Application for the Removal of Diclofenac Sodium From Aqueous Solution

**DOI:** 10.3389/fchem.2020.00274

**Published:** 2020-04-23

**Authors:** Nguyen Thi Minh Tam, Yun-guo Liu, Hassan Bashir, Peng Zhang, Shao-bo Liu, Xiaofei Tan, Ming-yang Dai, Mei-fang Li

**Affiliations:** ^1^College of Environmental Science and Engineering, Hunan University, Changsha, China; ^2^Key Laboratory of Environmental Biology and Pollution Control, Ministry of Education, Changsha, China; ^3^School of Metallurgy and Environment, Central South University, Changsha, China; ^4^School of Architecture and Art, Central South University, Changsha, China

**Keywords:** graphitic carbon, porous biochar, diclofenac sodium, potassium ferrate, lignin content, adsorption

## Abstract

Porous biochar containing graphitic carbon materials have received great attention from various disciplines, especially for environmental pollutant treatment, due to their cost-effective and specific textural properties. This study exhibited a two-step strategy to compose lignin-porous biochar containing graphitic carbon (LPGC) from pitch pine sawdust and investigated its adsorptive removal for diclofenac sodium (DCF) from an aqueous solution. Sulfuric acid (H_2_SO_4_) was utilized to obtain lignin content from biomass and potassium ferrate (K_2_FeO_4_) and was adopted to fulfill the synchronous carbonization and graphitization of LPGC. Through slow pyrolysis in atmospheric N_2_ (900°C – 2 h), the structure of the as-prepared sample was successfully modified. Using SEM images, a stripped layer structure was observed on the H_2_SO_4_-treated sample for both one-step and two-step activated samples, indicating the pronounced effect of H_2_SO_4_ in the layering of materials. K_2_FeO_4_ acted as an activator and catalyst to convert biomass into the porous graphitic structure. The BET surface area, XRD and Raman spectra analyses demonstrated that LPGC possessed a micro/mesoporous structure with a relatively large surface area (457.4 m^2^ g^−1^) as well as the presence of a graphitic structure. Further adsorption experiments revealed that LPGC exhibited a high DCF adsorption capacity (q_max_ = 159.7 mg g^−1^ at 298 K, pH = 6.5). The effects of ambient conditions such as contact time, solution pH, temperature, ionic strength, electrolyte background on the uptake of DCF were investigated by a batch adsorption experiment. Results indicated that the experimental data were best fitted with the pseudo second—order model and Langmuir isotherm model. Furthermore, the adsorption of DCF onto the LPGC process was spontaneous and endothermic. Electrostatic interaction, H-bonding interaction, and π-π interaction are the possible adsorption mechanisms. The porous biochar containing graphitic carbon obtained from the lignin content of pitch pine sawdust may be a potential material for eliminating organic pollutants from water bodies.

## Introduction

Over the past few decades, along with the development of medicine, the human healthcare products manufacturing industry has also grown to serve the demand of more than 7 billion people worldwide. Among the products, pharmaceuticals are widely used for preventing and treating diseases in humans and animals. It was estimated that the worldwide average per capita consumption of pharmaceuticals is around 15 g per year (Alder et al., 2006; Zhang et al., 2008). Environmentalists consider pharmaceuticals as emerging contaminants (ECs), which include various groups such as: analgesics, antibiotics, anti-inflammatories, regulatory blood lipid, and natural and synthetic hormones (Ahmed and Hameed, 2018). Because the conventional wastewater treatment plants are not specifically designed to remove pharmaceuticals and residues, they are almost unobstructed, flowing into water bodies and causing damage to animals and human beings (Alidina et al., 2014). It is especially dangerous for the environment and can even cause damage in marine life such as hormonal disorders and sex reversal in fish and amphibians. Many studies about the effect of ECs on the environment and human health have been conducted (Letzel et al., 2009; Michael et al., 2013; Alves et al., 2017; Batt et al., 2017; Benson et al., 2017). Among the pharmaceutical products, diclofenac (DCF) is known as the “world's most popular pain killer” and is listed as an environmental threat by the European Union (EU) and the US Environmental Protection Agency (USEPA) (Framework, 2013; Lonappan et al., 2016). DCF has been detected in water all over the world both in a low concentration (0.08 μg L^−1^) and a significant concentration (2.51 μg L^−1^) (Heberer, 2002) in wastewater effluents. It was reported that currently 0.1–8.3% of the total length of Europe's rivers could exceed the suggested EU Environmental Quality Standards for diclofenac (100 ng L^−1^) (Johnson et al., 2013). It is estimated that the world consumes about 1,443 ± 58 tons of DCF annually (Acuña et al., 2015), and hundreds of tons of it has been discharged into the environment. The contaminant is partly absorbed by the natural ecosystem and the rest blends into the marine environment. It was reported that even at very low concentrations, DCF can cause cytological alterations in rainbow trout (1 μg L^−1^), or tissue damage in several mussel species (250 μg L^−1^) (Ericson et al., 2010). Thus, DCF should be removed from wastewater by proper physicochemical treatment methods before disposal into the natural environment.

Various technologies such as adsorption, degradation, coagulation, oxidation degradation and so on have been applied in polluted water treatment. Among these methods, adsorption has the advantage of being easy to operate, is low cost, is highly efficient, has strong reproducibility, and is available as different adsorbents (Liu et al., 2019). Thus, it has been proven to be an effective and economically viable method of removing organic contaminants. One of the important materials used in adsorption techniques is biochar. Biochar is known as a type of solid carbonaceous material with profound properties (the presence of surface functional groups, moderate surface area, and porosity) which can effectively adsorb organic pollutants from the water environment. Many studies have been conducted to derive biochar from different types of biomass, such as shells, flowers, rice residues, bamboo, tobacco, sawdust, and sewage sludge, etc. Among the feedstocks, biochar derived from herbaceous plant materials is more favorable than others, due to its beneficial characteristics (e.g., abundant feedstock, easy to harvest, high potential to produce sustainable adsorbents and green supercapacitors; Tan et al., 2015). However, the raw biochar has limited ability to adsorb contaminants from aqueous solutions, especially for wastewater that contain high concentrations of pollution (Yao et al., 2013; Tan et al., 2016). Furthermore, biochar is an amorphous powder and is not easy to separate from the aqueous solution due to its small particle size (Tan et al., 2016). Thus, it is essential to enhance the biochar structure to improve its adsorption performance. For this reason, porous graphitic carbon materials (PGCs) have been developed and has increasingly attracted attention from various disciplines, especially in the field of environmental micro-pollutant treatment (Edathil et al., 2017; Siyasukh et al., 2018; Gupta et al., 2019; Shittu et al., 2019) and advanced materials for storing energy (Jiang et al., 2019; Kim et al., 2019; Xi et al., 2019; Xing et al., 2019). To obtain PGCs, researchers developed several methods such as catalytic activation of biomass (Sevilla and Fuertes, 2010; Wang et al., 2013; Navarro-Suárez et al., 2014) or sacrificial template methods using silica or surfactants (Gibot et al., 2016; Nita et al., 2016; Yan et al., 2017). The advantage of graphitic carbon over amorphous carbon materials is that the honeycomb structure facilitates the absorption of aromatic compounds including aromatic pharmaceuticals through π-π stacking. Nevertheless, the further development of PGCs fabrication process is limited by the consumption of expensive precursors and multiple time-consuming steps. It is therefore sensible to develop a low-cost, environmentally friendly, and effective approach to synthesize porous graphitic carbon.

The herbaceous plant biomass contains about 85–90% of cellulose, hemicellulose, and lignin, while organic extractives and inorganic minerals constitute the rest (Pasangulapati et al., 2012). Lignin, because of its aromatic molecule structure with a high degree of cross linking between the phenylpropane and β-O-4, presents an incentive feedstock for graphite production (Chatterjee et al., 2014a,b). This connection makes lignin more thermally stable than hemicellulose (Ramiah, 1970). In this study, we propose an approach for deriving a porous biochar containing graphitic carbon material from pitch pine sawdust. A graphitic structure was obtained through the structural separation of biomass components. Sulfuric acid (H_2_SO_4_) was employed to eliminate the cellulose/hemicellulose components in sawdust. Potassium ferrate (K_2_FeO_4_) is known as a strong oxidant reagent that can be directly applied in wastewater treatment without producing any polluting byproducts (Jiang et al., 2006; He et al., 2018). In addition, potassium ferrate was also utilized as an activated agent to modify the biochar structure (Wu J. et al., 2019). Zhang et al. (2019) used Fe—a transition metal—as a catalyst agent to form a graphitic structure of biochar while Demir et al. (2015) proposed graphitic biocarbon from the metal-catalyzed hydrothermal carbonization of lignin. To the best of our knowledge, the study on applications of potassium ferrate to convert lignin content of plant biomass is limited. In this study, K_2_FeO_4_ was utilized to simultaneously convert the lignin content of pitch pine biomass into a porous and graphitic structure. The nano composite was labeled as lignin-porous biochar containing graphitic carbon (LPGC). DCF was employed as a representative organic contaminant and its removal process from aqueous solution using LPGC was investigated.

Taking into account the advantageous characteristics of biochar and the necessity of removing DCF from the natural environment, the aims of our study were to: (1) prepare and characterize the LPGC; (2) explore the adsorption capacity of LPGC for DCF through studies of kinetics and isotherms; (3) investigate the impact of the DCF concentration, reaction temperature, solution pH, ionic strengths, and background electrolytes on the adsorption capacity of LPGC toward DCF; and to (4) examine the possible adsorption mechanism between adsorbent LPGC and adsorbate DCF.

## Materials and Methods

### Chemical Reagents

The pitch pine (*Pinus rigida*) sawdust, a byproduct of the wood manufacturing industry was utilized in this study. The chemical compositions of cellulose, hemicellulose, and lignin in pitch pine wood are 46, 24, and 27%, respectively (Rangabhashiyam and Balasubramanian, 2019). The biomass used was obtained from a furniture factory located in Changsha (Hunan, China). Diclofenac sodium salt (purity 99%) was purchased from Shanghai Yien Chemical Technique Co., Ltd. The chemical structure and physicochemical properties of DCF are described in the Table S1. The other chemical reagents used were supplied by Shanghai Macklin Biochemical Co., Ltd. The water used in all experiments was prepared in Milli-Q water (18.25 MΩ.cm at 25°C) obtained from a Millipore water purification system. All chemicals used in this study were of analytical grade.

To prepare DCF stock solution (5 g L^−1^), 0.5 g DCF solid (99% purity) was dissolved in 10 ml pure methanol before continuously being mixed into deionized water (90 ml). The solution was then stored in an amber colored bottle at 4 ± 1°C for later use. For each experiment, different working solutions were derived by diluting the as-prepared solution with deionized water.

### Synthesis of Lignin Biochar Containing Graphitic Carbon and Other Compared Materials

**Figure 1:**
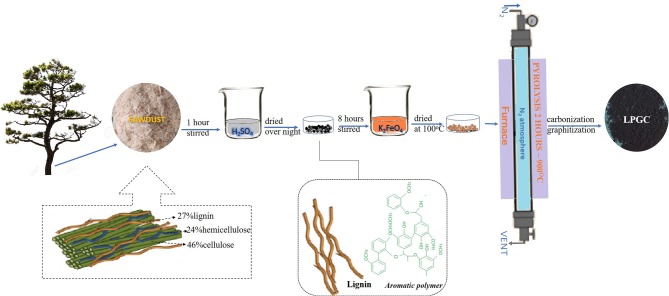
Schematic diagram of the preparation of LPGC.

First, pitch pine sawdust was mixed in H_2_SO_4_ (72%) solution at a ratio of 10% w/v to obtain the lignin component of the biomass. The mixed solution was stirred magnetically at room temperature for 1 h to eliminate cellulose and hemicellulose components. The solution concentration was then adjusted to 4% by deionized water and kept at 120°C for 1 h. After that, 0.45 μm filter paper was used to refine the slurry. The sample was washed several times to remove the excess chemicals and dried overnight at 60°C. After evaporating, we obtained a solid sample. The solid sample was then crushed into powder and dispersed in 0.1 M K_2_FeO_4_ solution (100 ml) and continuously stirred in 8 h and dried overnight at 100°C to obtain a solid mixture. The mixture was then transferred into a tube furnace and heated at 900°C for 2 h with a heating rate of 5°C min^−1^ in atmospheric N_2_. Ultimately, the sample was collected and washed with deionized water, followed by drying at 80°C. The resultant sample was denoted as LPGC. The preparation process is schematically illustrated in Figure 1.

For comparison, three different samples were prepared. A H_2_SO_4_-treated sample was made by mixing sawdust with an H_2_SO_4_ (72%) solution at a ratio of 10% of w/v and stirred continuously for 1 h. The concentration of H_2_SO_4_ was adjusted to 4% by adding deionized water. The sample was incubated at 120°C for 1 h then filtered by 0.45 μm filter paper and dried overnight at 60°C to obtain a solid mixture. At the same time, a K_2_FeO_4_-treated sample was prepared by adding 3.0 g of sawdust to the K_2_FeO_4_ solution (100 ml, 0.1 M) and continuously stirred for 8 h, then dried overnight at 100°C. Subsequently, a similar pyrolysis process as described above was carried out with the H_2_SO_4_-treated sample, K_2_FeO_4_-treated sample, and no-treated sample. All the samples were transferred into a tube furnace and heated at 900°C for 2 h with a heating rate of 5°C min^−1^ in atmospheric N_2_. The resultant H_2_SO_4_-treated, K_2_FeO_4_-treated, and no-treated samples were collected and labeled as lignin biochar (LBC), cellulose/lignin biochar (C/LBC), and pristine biochar (BC), respectively.

### Characterizations

The morphological studies of the samples were carried out by scanning electron microscopy (SEM) (S4800, Hitachi, Japan) and transmission electron microscopy (TEM) (Tecnai G2 20, FEI, USA). The elemental composition of samples was observed with energy-dispersive X ray spectroscopy (EDS) (Genesis, Edax, USA). To obtain a X-ray diffraction (XRD) pattern of the samples, a Bruker AXS D8 Advance diffractometer was used with Cu Kα radiation (acceleration voltage of 40 kV, 2 theta interval 0.02°, scan speed 0.1 sec/step, λ = 0.15418 nm). The Raman spectra was obtained from a LabRAM HR UV Raman spectrometer at the laser excitation wavelength of 532 nm and power 10 mV. The spectrum was measured at room temperature in the spectral range of 4,000–400 cm^−1^. The BET surface areas and pore structure of the samples were investigated by Nitrogen adsorption-desorption method temperature of liquid nitrogen (Quantachrome Instruments Quadrasorb EVO, USA). The surface chemistry and chemical state of elements were analyzed by X-ray photoelectron spectroscopy (XPS) (ESCALAB 250Xi, Thermo Fisher, USA) under Al- Kα (hv = 1486.6 eV), power 150 W, 500 μm beam spot. FT-IR studies were performed by a spectrophotometer (IR Tracer-100, Shimadzu, Japan) in the wavenumber range of 400–4,000 cm^−1^. For zeta potential analysis, 25 mg of adsorbents was added to 25 ml Milli-Q water and the pH was adjusted in the range of 2.0–10.0 with 0.1 M NaOH or HCl. Zeta potential meter (Zetasizer Nano-ZS90, Malvern Instruments, Malvern, UK) was used to perform the results.

### Adsorption Experiments

For adsorption experiments, DCF solution with a concentration of 20 mg L^−1^ was chosen and derived by diluting stock solution into deionized water. NaOH and HCl with negligible volumes were used to adjust the pH to desired values. In all batch sorption experiments, 0.005 g of adsorbents was added in conical flasks (volume 100 ml) with 50 ml DCF solution (20 mg L^−1^). The conical flasks were put in a rotary thermostatic oscillator and shaken at a constant speed (160 rpm) at a temperature of 25°C for 24 h and then the supernatants were collected by filtration with 0.45 μm membrane filters.

The concentration of DCF in both the initial and adsorbent solutions were examined by an UV-VIS spectrophotometer (UV-2550, Shimadzu, Japan) at the wavelength of the highest absorbance 276 nm (Bhadra et al., 2016). A linear coefficient was obtained from the calibration curve of DCF and used to convert the absorbance to the concentration through Equation 1 (Rosset et al., 2019).

(1) $$\begin{array}{r} S=\frac{A}{a} \end{array}$$

Where: S is the solution concentration (mg L^−1^); A is the absorbance at wavelength 276 nm; a is the linear coefficient of the calibration curve.

The adsorbed DCF amount was calculated by the difference between the sorption solution concentrations at initial time and equilibrium time. The adsorption capacities were obtained by the following equation:

(2) $$\begin{array}{r} q_{e}=\frac{(C_{o}-C_{e})V}{m} \end{array}$$

where C_o_ is the initial and C_e_ is the equilibrium concentration of DCF (mg L^−1^), V and m are the volume of the solution (L), and adsorbents doses (g), respectively.

DCF adsorption kinetic experiments were carried out to determine the minimum time required to reach adsorption equilibrium state. Fifty microgram of adsorbents was added to 50 ml of DCF 20 mg L^−1^ solution at pH ≈ 6.5 and 298 K. All samples were then shaken for different time intervals (ranging from 0.17 to 24 h). Two models, Pseudo first-order and Pseudo second-order were used to evaluate the adsorption process. The equations of each model are given as Equation (1s) and (2s) in the Supplementary Materials.

To obtain information about the maximum adsorption capacities, DCF adsorption isotherm experiments were conducted at pH ≈ 6.5 under three different temperatures (298, 308, and 318 K). The initial concentration of the DCF solution ranged from 5 to 25 mg L^−1^. Two isotherm models, namely Langmuir isotherm and Freundlich isotherm, were used to simulate the adsorption process. The isotherm model equations are given as Equation (3s) and (4s) in the Supplementary Materials.

A thermodynamics study can reveal information on the inherent energy change of adsorbent and the adsorption mechanism between adsorbent and adsorbate (Sun et al., 2014; Jiang et al., 2015). The effects of temperature on adsorption were explored through some thermodynamic parameters, such as Gibbs free energy of adsorption (ΔG°), heat of the adsorption (ΔH°) and standard entropy changes (ΔS°) (Suriyanon et al., 2013). Here, the thermodynamics adsorption of DCF on LPGC was carried out at various temperatures (298, 308, and 318 K), 50 mg LPGC was added into the 20 mg L^−1^ DCF solution (50 ml) at pH ≈ 6.5, contact time 24 h. Thermodynamic parameters calculation equations are given as Equation (5s) and (6s) in the Supplementary Materials.

To investigate the influence of pH onto the adsorption process, batch experiments with the same conditions of kinetic experiments (excluded pH value) were conducted. The range of initial pH was from 2.0 to 10.0, and 0.1 M NaOH or 0.1 M HCl with negligible volumes were used to adjust the pH of the solution.

NaCl with different concentrations varying from 0.001 to 0.1 M were used to explore the effect of ionic strength on the removal of DCF from the aqueous solution by as-prepared materials. The experiment conditions were the same as the kinetic experiment conditions.

Along with other ambient factors, background electrolyte ions also have an impact on the adsorption process. To examine the effect of this factor onto DCF adsorption by LPGC, several background electrolyte ions such as Na^+^, K^+^, Mg^2+^, Ca^2+^, NO${}_{3}^{-}$, SO${}_{4}^{2-}$, and PO${}_{4}^{3-}$ were used. The electrolyte ions with three different concentrations (0.001, 0.01, and 0.1 M) were added into 50 ml DCF solution (20 mg L^−1^) containing 50 mg LPGC at 298 K and pH ≈ 6.5, respectively.

## Results and Discussion

### Characterization of Adsorbents

#### Surface Morphology Analysis

**Figure 2:**
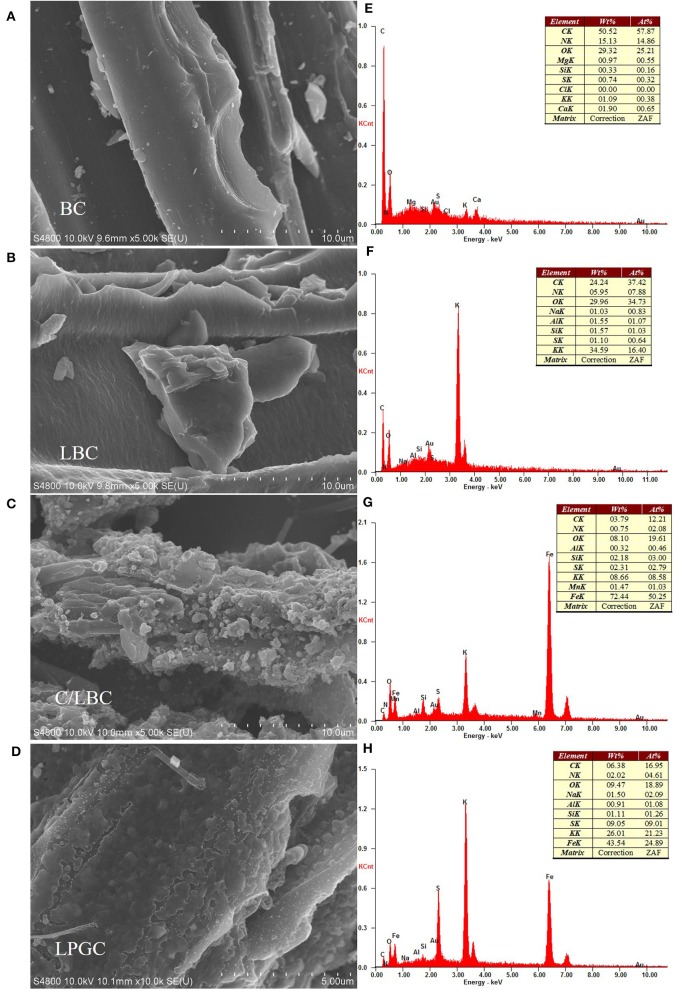
Scanning electron microscopy (SEM) images of **(A)** BC, **(B)** LBC, **(C)** C/LBC, and **(D)** LPGC, and the corresponding EDS image of **(E)** BC, **(F)** LBC, **(G)** C/LBC, and **(H)** LPGC.

**Figure 3:**
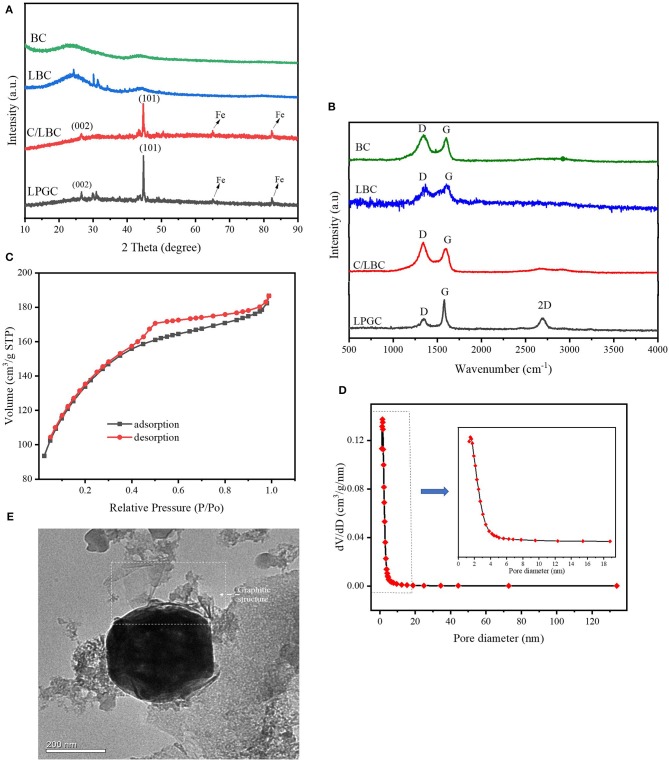
**(A)** XRD patterns and **(B)** Raman spectra of LPGC and other samples; **(C)** N_2_ adsorption-desorption isotherm, **(D)** pore size distribution, and **(E)** transmission electron microscopy (TEM) image of LPGC sample.

The combination of the scanning electron microscope (SEM) and energy-dispersive X-ray spectroscopy (EDS) provides a powerful tool for exploring the morphological, topographical information of the biochar, while transmission electron microscopy (TEM) can reveal the structural quality and the layer state of material. The SEM images of BC, LBC, C/LBC, and LPGC are shown in Figures 2A–D. The EDS images of as-prepared samples are displayed in Figures 2E,F. The TEM image of LPGC is presented in Figure 3E. As can be observed, there are many nanoparticles on the surface of LPGC and C/LBC compared with BC. The original surface of BC possessed a relatively smooth surface with a plate-like morphology (Figure 2A). After one step modification, the H_2_SO_4_-treated sample (LBC) obtained a stripped layer structure with small and even ripples (Figure 2B). Meanwhile the morphology was totally changed for the K_2_FeO_4_-treated sample (C/LBC). Its surface became rough and uneven with various lumps of different diameters (Figure 2C). Figure 2D displays the outer shape of LPGC—the two-step modification sample. Compared with the previous samples, LGBC exhibited a different surface structure with very high roughness and many parallel stripped scratches. In addition, it is easy to recognize that many solid particles appeared on the surface of C/LBC and LPGC, which confirmed the loading of iron particles. Moreover, the EDS results (Figures 2G,H) show that 72.44 and 43.45 wt% of Fe were found in C/LBC and LPGC, respectively. It could be concluded that Fe molecules successfully attached on to the samples treated with K_2_FeO_4._ Furthermore, the TEM image of LPGC (Figure 3E) indicated the presence of graphitic carbon.

#### XRD and Raman Analysis

The composition and crystalline phase of the as-prepared samples were investigated by XRD and Raman spectra. The XRD patterns of samples are shown in Figure 3A. There was a broad peak in BC and some characteristic peaks at θ values of 24.2°, 30.1°, and 31.3° in LBC, which could be ascribed to amorphous carbon (Tseng et al., 2011; Jiang et al., 2018), while in the LPGC and C/LBC these peaks are weaker, indicating the significant changes of their carbon characteristic. Furthermore, it can be observed that two diffraction peaks at θ values of 26.6° and 44.7° in C/LBC were obviously higher in LPGC, which can be indexed to typical (002) and (001) reflection of graphitic carbon (JCPDS No. 41-1487) (Gong et al., 2017). New diffraction peaks at 65.0° and 82.3° can also be seen in both C/LBC and LPGC, indicating the presence of Fe (JCPDS No. 06-0696) (Jiang et al., 2018). Furthermore, some peaks at θ value between 40° and 50° that corresponded to cementite (Fe_3_C) were observed in the XRD pattern of C/LBC and LPGC (Yan et al., 2018). It again confirmed that Fe nanoparticles were successfully loaded on the surface of two K_2_FeO_4_-treated materials (C/LBC and LPGC) during the synthesis process. Additionally, the peaks between 30° and 35° in LPGC could be attributed to iron oxide bands (JCPDS No. 19-0629) (Macías-Martínez et al., 2016; Atul et al., 2019). The presence of all the peaks in the LPGC XRD pattern showed the formation of the desired porous graphitic carbon. In addition, the graphitic structure was also demonstrated by Raman spectroscopy. As can be seen in Figure 3B, two peaks were present which belong to the D-band and G-band in the Raman spectra of all samples. Basically, the D-band (near 1,350 cm^−1^) exhibits additional first-order bands, corresponding to defect sites or disordered sp2-hybridized carbon atoms of graphite and the G-band (near 1,580 cm^−1^) associated with the zone-center phonon of sp2-bonded carbon atoms (Gong et al., 2017; Li et al., 2018). In LPGC Raman spectra, the intensity of the D-band was lower than other samples, it may be attributable to the fact that the amorphous structure in LPGC was reduced, and instead there was the formation of another structure. In addition, in comparison with other samples, it was notable that Raman spectra of LPGC showed a peak at the wavenumber of 2,685 cm^−1^, which corresponds to the 2D-band-a signature of the graphitic carbon (Li et al., 2018). This indicated that the Raman spectrum of LPGC is referring to its graphitic portion. Generally, the intensity ratio of the D-band to G-band (I_D_/I_G_) was used as an useful parameter to determine the degree of order or disorder in crystalline structures of carbonaceous materials (Sadezky et al., 2005). The I_D_/I_G_ values of LPGC were relatively low (0.48) compared with that of C/LBC (1.07) and BC (1.22). In short, the XRD and Raman spectra analysis confirmed that after two-step modification, the LPGC was successfully converted into porous biochar containing graphitic carbon.

#### N_2_ Adsorption-Desorption Isotherms

N_2_ adsorption-desorption isotherms and the porosity distribution of LPGC are shown in Figure 3C. Based on the International Union of Pure and Applied Chemistry (IUPAC) classification, the adsorption/desorption isotherm of LPGC belongs to Type-IV isotherm. It can be seen that the isotherm curve showed a hysteresis loop in the relative vapor pressure range of 0.4–1.0 bar, which demonstrated the presence of a hierarchical micro/mesoporous system on the surface of LPGC (Abo El Naga et al., 2019). This was further confirmed by the Barrett-Joyner-Halenda (BJH) pore-size distribution result. As can be observed in Figure 3D, the pore sizes of LPGC were allocated in a range of 1–133 nm, focused between 1.6 and 18.7 nm. The N_2_ adsorption with the pore diameter distribution curves of C/LBC and BC are shown in Figure S1. The corresponding porosity data of LPGC and comparison samples are listed in Table 1. After a process of pyrolysis at 900°C, the BET surface area of LPGC was 457.4 m^2^ g^−1^, while those of C/LBC and BC were 175.8 and 426.7 m^2^ g^−1^, respectively. Kim et al. (2012) carried out research on the influence of pyrolysis temperature on physicochemical properties of biochar obtained from the pyrolysis of pitch pine (*Pinus rigida)*. The study reported that at different pyrolysis temperatures, the *Pinus rigida* sawdust derived biochar possessed different BET surface areas with an increasing trend (2.9, 4.8, and 175 m^2^ g^−1^, corresponding with the pyrolysis temperature at 300, 400, and 500°C, respectively). Tan et al. (2015) also confirmed that pyrolytic temperature plays a significant role in changing biochar characteristics. In this study, the BC sample without chemical activation obtained a relatively high BET surface area (426.7 m^2^ g^−1^), which might be due to the influence of a high pyrolysis temperature. Furthermore, it can be seen from the SEM images (Figures 2C,D), that plenty of nano particles appeared on the LPGC and C/LBC surface, which might block the small pores on their surfaces. In addition, in Figure 3C there is the appearance of an “ink bottle” between the isotherm adsorption—desorption curves of LPGC. It can be hypothesized that there were micropores inside of mesopores resulting in the process of N_2_ desorption, thus, after N_2_ liquid in the mesopores desorbed, the N_2_ gas bound in the micropore would suddenly escape. By analyzing the BET results, the surface area of the C/LBC (one-step activated) was significantly decreased while the surface area of LPGC (two-step activated) insignificantly increased, compared with the BET surface of no-treated biochar (BC).

**Table 1:** Parameters describe the surface area and pore structure of LPGC and comparison samples.

**Samples**	**Isotherms type**	**BET surface area (m^**2**^ g^**−1**^)**	**Total pore volume (cm^**3**^ g^**−1**^)**	**Micropore volume (cm^**3**^ g^**−1**^)**	**Mesopore volume (cm^**3**^ g^**−1**^)**	**Ratio of mesopore on total pore volume (%)**	**Average pore diameter (nm)**
LPGC	IV	457.4	0.288	0.143	0.145	50.3	2.524
C/LBC	IV	175.8	0.141	0.049	0.092	65.2	3.214
BC	I	426.7	0.265	0.154	0.111	41.8	2.449

#### XPS Analysis

**Figure 4:**
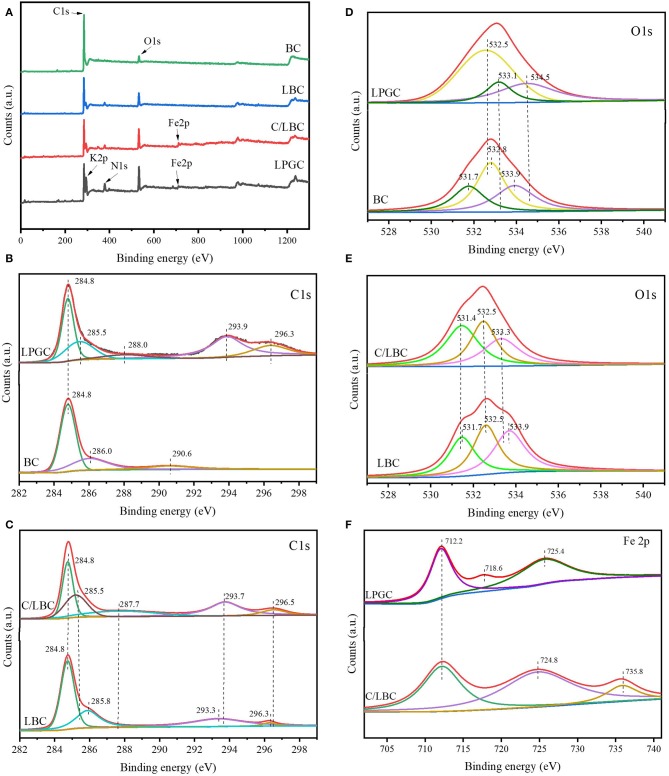
**(A)** XPS survey spectra of LPGC and other samples; H-R C 1s spectrum of **(B)** LGBC and BC, **(C)** C/LBC, and LBC; H-R O 1s spectrum of **(D)** LPGC and BC, **(E)** C/LBC, and LBC; **(F)** H-R Fe 2p spectrum of LPGC and C/LBC.

To explore the surface chemical compositions of the as-prepared samples, X-ray photoelectron spectroscopy (XPS) was applied. Figure 4A shows the survey spectra of LPGC, C/LBC, LBC, and BC samples. It can be observed that C, O, N, K, and Fe in the XPS spectrum of LGBC and C/LBC were present, which is consistent with the EDS characterization results. Figures 4B–E shows the high-resolution (H-R) C 1s and O 1s XPS spectra of as-prepared samples. The H-R XPS spectrum of Fe 2p for LPGC and C/LBC are presented in Figure 4F.

Regarding the C 1s, as can be seen in Figures 4B,C, the peak located at 284.8 eV corresponded to the C-C bonds with a sp^2^ hybridization zone and appeared in all samples. The peak at 285.5 eV was associated with the contribution of both C-O and C-OH functional groups (Rojas et al., 2016) and can only be observed at LPGC and C/LBC. There were other peaks at 287.7, 293.7, and 296.5 eV in the C/LBC H-R C 1s XPS spectrum which can be ascribed to C-O, C=C, O=C-O, and π-π^*^ “shake-up” satellites characteristic of graphite-like carbon (Chang et al., 2019). Those peaks then slightly shifted to higher binding energy in the C 1s spectrum of LPGC (288.0, 293.9, and 296.5 eV). This finding suggested that the changes in electron cloud density in the carbon atoms, were likely due to the more graphitic structure of LPGC compared with C/LBC. For the O 1s, XPS spectra for all samples are shown in Figures 4D,E. There was a peak at 531.7 eV in BC and LBC, which represented surface OH groups (Lu et al., 2018), but it did not present in C/LBC and LPGC, instead, there was the presence of a peak at 532.5 eV (in both LPGC and C/LBC). This peak can be attributed to the signal of C-O/C=O bonds with a larger contribution from C-O bonds than from C=O bonds. The next peaks at 533.1 eV in LPGC and 533.3 eV in C/LBC corresponded to C-OH bonds, while the peak at 534.5 eV, only seen in LPGC-, was associated with oxygen in water molecules (Rojas et al., 2016). Additionally, the H-R XPS spectrum for Fe 2p of LPGC (Figure 4F) shows two peaks around the binding energy of 712.2 and 725.4 eV, which were ascribed to Fe 2p_3/2_ and Fe 2p_1/2_, respectively (Gao et al., 2017), and the peak at 718.6 eV was attributed to the satellite peak of Fe (Li et al., 2019). For C/LBC, the peak at 712.2 eV was also observed on the Fe 2p spectrum, while the peaks at 724.8 eV could be assigned to Fe 2p_1/2_ (Li et al., 2019) and the peak at 735.8 eV presented a contribution of Fe^3+^ (Peña et al., 2019). The XPS results indicated that the surface of LPGC was significantly changed by using a two-step modification process. The XPS of BC and LBC only showed the existence of the sp^2^ hybridization zone, while multiple polar functional groups could be observed on the C/LBC and LPGC surface which might contribute to the formation of a porous graphitic structure of these samples.

### DCF Adsorption Kinetics

**Figure 5:**
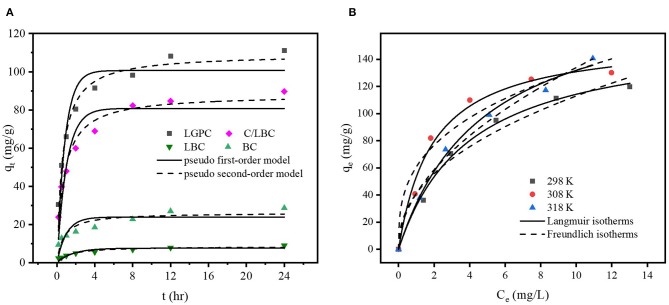
**(A)** Effect of contact time on the adsorption of DCF by LPGC and three comparison samples and **(B)** adsorption isotherms of DCF by LPGC at three different temperatures.

Adsorption kinetics display a strong relationship with the physical and/or chemical characteristics of the biochar (Tan et al., 2015). The adsorption kinetics of DCF onto LPGC, C/LBC, LBC, and BC are displayed in Figure 5A. The uptake of DCF onto LPGC and C/LBC increased rapidly in the first 4 h, then gradually increased from 4 to 12 h, while the adsorption capacity of LBC and BC were insignificant. The maximum adsorption capacity was reached at about 24 and 20 h for LPGC and C/LBC, respectively; and 8 h for LBC and BC. Twenty-four hours was therefore chosen as the reaction time, while LPGC and BC were chosen as absorbents in further adsorption experiments.

To further understand the mechanism of adsorption, two conventional kinetic models (Pseudo first-order and Pseudo second-order) were used to analyze and simulate the experiment results of DCF adsorption. The model descriptions are given in the Supplementary Materials. The kinetic parameters and correlation coefficients (*R*^2^) of two kinetic models are listed in Table 2. As can be seen, the determined coefficients of the Pseudo second-order model were higher than those of the Pseudo first-order model for all samples. In addition, the simulated adsorption capacity results, which were provided by the Pseudo second-order model, were very close to the experimental data. It further confirmed the feasibility of the Pseudo second-order model to fit the kinetic DCF adsorption results. The trend indicated that the chemisorption might be the primary mechanism for the uptake of DCF onto LGBC and other samples. It is worth noting that the DCF adsorption capacity of K_2_FeO_4_-treated samples (112.1 and 91.3 mg g^−1^ corresponding to LPGC and C/LBC) are much higher than those of non- K_2_FeO_4_-treated samples (9.2 and 28.3 mg g^−1^ corresponding to LBC and BC). Furthermore, the results of the characterization of adsorbents confirmed the presence of Fe iron particles on the LPGC and C/LBC surface. Thus, Fe plays an important role in the removal efficiency of LPGC and C/LBC toward DCF, and the effect of Fe-based technologies for pharmaceutical removal from water is further discussed in a study by Liu et al. (2016).

**Table 2:** Kinetic parameters adsorption of DCF on LPGC and comparison samples.

**Absorbents**	**q_**e, exp**_ (mg g^**−1**^)**	**Pseudo-first-order model**			**Pseudo-second-order model**		
		**q_**e, 1**_ (mg g^**−1**^)**	**k_1 (1 h^−1^)_**	***R*^**2**^**	**q_**e, 2**_ (mg g^**−1**^)**	**k_**2**_ (g mg^**−1**^ h^**−1**^)**	***R*^**2**^**
LPGC	111.09	105.4	1.17	0.90	112.1	0.015	0.98
C/LBC	89.66	85.3	0.97	0.87	91.3	0.015	0.96
LBC	9.00	8.3	0.59	0.80	9.2	0.091	0.90
BC	28.75	26.2	1.00	0.63	28.3	0.053	0.80

*Experimental conditions: m/V = 0.1 mg/mL, C_o_ = 20 mg L^**-**1^, T = 298 K, pH = 6.5*.

### DCF Adsorption Isotherm

To explore the adsorption phenomena in the liquid phase at equilibrium, adsorption isotherm experiments were conducted. Figure 5B shows the uptake of DCF by LPGC at three different temperatures and Figure S2 illustrates the DCF adsorption by BC at three temperatures (298, 308, and 318 K, respectively). As can be seen, the uptake of DCF onto LPGC significantly increased with low initial DCF concentration. It might be the result of numerous active sites located on the surface of the adsorbent, which was easy to access. However, when the concentration of the DCF solution increased, the adsorption capacity of LPGC tended to decrease. This revealed that the active sites became fewer when the reaction state was near the equilibrium point. Two adsorption isotherm models, namely Langmuir and Freundlich, were used to investigate the mechanism of the adsorption equilibrium. Theoretical aspects regarding adsorption isotherm models are given in the Supplementary Materials. The relevant results simulated from those equations are listed in Table 3. The Langmuir parameter q_max_ corresponds to the monolayer's maximum adsorption capacity, while the Freundlich constant K_F_ roughly reflects the adsorption capacity for comparing multiple adsorbent-adsorbate systems (Tong et al., 2019). Results in Table 3 indicate that the Langmuir model exhibited a higher correlation coefficient value than the Freundlich model and it could be assumed that the DCF adsorption by LPGC was a monolayer reaction, which may occur on homogeneous adsorption sites. Furthermore, the adsorption isotherm results of BC were fitted to both models with an insignificant difference between the *R*^2^ values of the Langmuir and Freundlich models. It was also reported that q_max_ tended to be related with the reaction temperature; the higher the q_max_ value, the higher the heat of sorption. This could be the base for the formation of stronger bonds (Ofomaja et al., 2010). In this study, the simulated maximum adsorption capacity (q_max_) of the Langmuir model increased when experimental temperature increased (from 298 to 318 K), which indicated that a higher temperature could be more favorable for the uptake of DCF onto LPGC.

**Table 3:** Isotherm model parameters of DCF adsorption by LPGC and BC at three temperatures.

**Adsorbents**	**Temperature (K)**	**Langmuir**			**Freundlich**		
		**q_**max**_ (mg/g)**	**K_**L**_ (L/mg)**	***R*^**2**^**	**K_**F**_ [(mg/g)/(mg/L)^**1/n**^]**	***n***	***R*^**2**^**
LPGC	298	159.7	0.25	0.98	41.11	0.44	0.91
	308	165.9	0.52	0.96	60.72	0.33	0.83
	318	195.1	0.21	0.98	41.08	0.51	0.97
BC	298	35.2	0.36	0.93	14.32	0.26	0.96
	308	37.4	0.33	0.96	14.55	0.28	0.98
	318	46.3	0.30	0.98	16.84	0.29	0.99

*Experimental conditions: m/V = 0.1 mg/mL, C_o_ ranged 5–25 mg L^−1^, T = 298, 308, 318 K, pH = 6.5, t = 24 h*.

### DCF Adsorption Thermodynamic

The thermodynamic parameters were calculated from the data in Figure 5B by Equation (5s) and (6s) (Supplementary Materials). The results are shown in Table 4. The values of ΔG° were found to be negative at all temperatures, which revealed that the whole process was spontaneous. The positive value of ΔH° implied that the adsorption reaction was an endothermic process, which was suggested by the experimental results that the adsorption capacity of DCF increased when the temperature was raised. Furthermore, the positive value of ΔS° suggested that the randomness of the adsorbate-adsorbent interface could be increased during the reaction process.

**Table 4:** Thermodynamic parameters of DCF adsorption on LPGC.

**T (K)**	**lnK^**0**^**	**ΔG^**°**^ (kJ mol^-1^)**	**ΔS^**°**^ (J K^-1^ mol^-1^)**	**ΔH^**°**^ (kJ mol^-1^)**
298	1.22	−3.02	0.015	1.562
308	1.36	−3.50		
318	1.26	−3.34		

*Experimental conditions: m/V = 0.1 mg/mL, C_o_ = 20 mg L^−1^, T = 298, 308, 318 K, pH = 6.5, t = 24 h*.

### Impact of Solution pH

**Figure 6:**
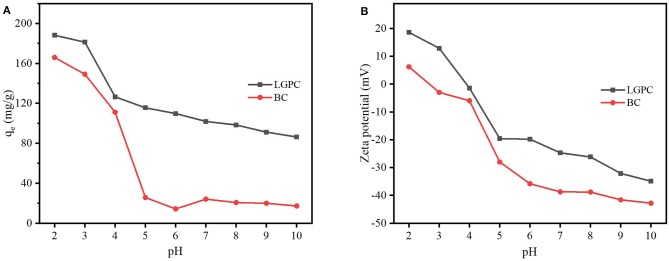
**(A)** Effects of solution pH on DCF adsorption by LPGC and BC and **(B)** zeta potential of LPGC and BC at different solution pH.

The value of solution pH is an important parameter for investigating the adsorption mechanism, since adsorption is a surface-controlled process and the variation of the pH value can change the surface charge of the adsorbent or the allocation of adsorbate in solution as well. Figure 6A shows the impact of initial pH solutions on DCF adsorption by LPGC and BC with a pH value ranging from 2.0 to 10.0. The results showed that the adsorption capacity was relatively high when the pH value was 2 and 3. It was then dramatically decreased at pH = 4.0 with LPGC and pH = 5.0 with BC. This phenomenon was consistent with the results of zeta potential (Figure 6B). The zeta potential value of LPGC and BC changed from positive to negative when the pH value increased. It demonstrated a negative charge on the LPGC surface over a wide range of pH values, and this surface charged value decreased with the increase of pH values. The zero point of charge (pH_zpc_) was found to be equal to 4.0, and at this point the LPGC surface became negatively charged. The DCF molecules could then be isolated to the anionic form, creating an electrostatic repulsive interaction between the negative charge of DCF anions and LGBC, which may result in a lower adsorption capacity of DCF onto adsorbents (Figure 6A). In addition, it was reported that the solubility of DCF was low (<2.37 mg/L) at pH values lower than its pK_a_ (4.15), indicating a presence of DCF in its neutral form (Bhadra et al., 2016; Larous and Meniai, 2016). It might explain the decreasing trend in adsorption capacity when the pH value increased above 4.2 (pH>pK_a_), since the solubility of DCF became higher.

### Impact of Ionic Strength

**Figure 7:**
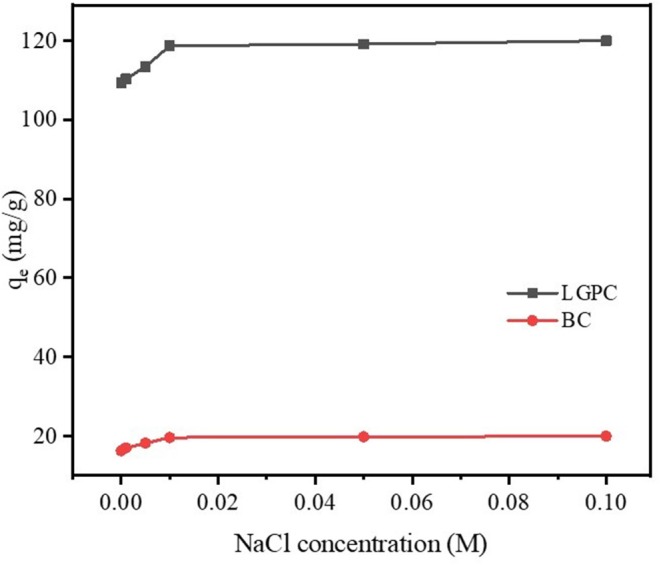
Effect of the ionic strength on DCF adsorption by LPGC and BC.

It was reported that wastewater contains not only organic contaminants but also various salts with ionic strengths, which may influence the elimination of contaminants (Xu et al., 2012). NaCl was chosen to explore the impact of ionic strength on the removal of DCF by LPGC due to its widespread presence in wastewater as well as other water sources. A series of experiments were conducted by adding different concentrations of NaCl into DCF compiled adsorbents (LPGC and BC), the results of which are shown in Figure 7. As can be seen, DCF adsorption capacity was improved in the presence of NaCl, and it was more obvious with adsorbent LPGC (q_e_ increased 8.83% when added NaCl 0.1 M into the solution). The enhanced coefficient activity of hydrophobic organic compounds that resulted in their solubility reduction, might be due to the increase in ionic strength, which was beneficial for the adsorption process (Zhang et al., 2010). In addition, Wu L. et al. (2019) used NaCl 10 mM as the background ionic strength during their experiment to enhance adsorption capacity of adsorbents, confirming its favorable role for DCF adsorption.

### Impact of Background Electrolyte

**Figure 8:**
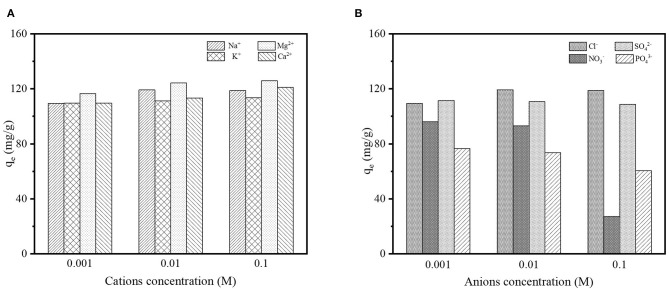
**(A)** Effect of background electrolyte cations and **(B)** effect of background electrolyte anions on DCF adsorption by LPGC.

Several types of background electrolyte ions such as Na^+^, K^+^, Mg^2+^, Ca^2+^, Cl^−^, NO${}_{3}^{-}$, SO${}_{4}^{2-}$, and PO${}_{4}^{3-}$ were simultaneously presented with contaminants in wastewater, which may influence the removal efficiency of contaminants from wastewater effluents. Thus, the impact of those background electrolytes on DCF adsorption was studied in the presence of cations (Na^+^, K^+^, Mg^2+^, and Ca^2+^) and anions (Cl^−^, NO${}_{3}^{-}$, SO${}_{4}^{2-}$, and PO${}_{4}^{3-}$) at three different concentrations (0.001, 0.01, and 0.1 M) for each type of ion; the results are shown in Figure 8. As can be seen in Figure 8A, the cations supported the adsorption process and the adsorption capacity increased with higher concentrations of cations. It may be due to the fact that the surface charge of DCF molecules at the studied pH (≈ 6.5) was negative, which led to an electrostatic interaction between the cations and the negatively charged DCF. On the contrary, the anions might hinder the adsorption of DCF onto LPGC (Figure 8B). Obviously, the adsorption capacity significantly declined in the presence of anions NO${}_{3}^{-}$ and PO${}_{4}^{3-}$. The phenomena might be mainly attributed to the occupation of NO${}_{3}^{-}$ and PO${}_{4}^{3}$ for limited adsorption sites on the LPGC surface, which resulted in a decrease in its adsorption capacity.

### Comparisons Between Studied Materials and Different Biomass-Derived Adsorbents

Table 5 provides an overview of different biomass-derived adsorbents used for DCF adsorption in terms of adsorption capacity. A straightforward comparison may not be objective since the experimental condition and the synthesized methodologies were different. Though, from the perspective of the efficiency of adsorbents, the comparison may provide a perception of the performance of a porous biochar containing graphitic carbon, obtained from the lignin content of pitch pine biomass (LPGC). From Table 5, it can be shown that the LPGC presents a significant adsorption capacity for DCF compared to the other biomass derived adsorbents such as pine wood, pig manure, Isabel grape bagasse, potato peel, cocoa shell, and tea waste, etc. However, the performance of LPGC on DCF adsorption was weaker than some other adsorbents such as rice straw (277.78 mg g^−1^) or sugarcane bagasse (315.0 mg g^−1^). Nevertheless, the dosage of LPGC used in adsorption experiments was small compared with rice straw and sugarcane bagasse. Therefore, LPGC can still be evaluated as a potential adsorbent for the efficient removal of DCF from wastewater.

**Table 5:** Comparison of DCF adsorption capacity with other biochar derived adsorbents.

**Material**	**BET surface area (m^**2**^/g)**	**Adsorption ambient conditions**				**Maximum adsorption capacity (mg g^**−1**^)**	**References**
		**pH**	**Temperature (K)**	**Adsorbent dose (g)**	**Initial concentration of DCF solution (mg L^**−1**^)**		
Rice straw	287.8	7	293	0.3	15.9	277.8	Xia et al., 2019
Pinewood	13.3	6.5	298	2	0.5	1.1	Lonappan et al., 2019
Pig manure	43.5	6.5	298	2	0.5	4.1	Lonappan et al., 2019
Sugarcane	1145.0	2	298	0.4	50.0	315.0	Abo El Naga et al., 2019
Isabel grape	2.0	5	295	0.0005	10.0	11.1	Antunes et al., 2012
Potato peel	866	5	–	10	50.0	68.5	Bernardo et al., 2016
Cocoa shell	619	7	298	2.5	100.0	63.5	Saucier et al., 2015
Tea waste	865.4	6.47	–	0.3	30.0	62.5	Malhotra et al., 2018
LPGC	457.4	6.5	298	0.05	20.0	159.7	This study
C/LBC	175.8	6.5	298	0.05	20.0	91.3	This study
BC	426.7	6.5	298	0.05	20.0	35.2	This study

### Possible Mechanisms

DCF is known as a hydrophobic compound with a weak acidity (pKa is around 4.15). As can be seen in section Impact of Solution pH, the higher adsorption capacity of LPGC was observed at an acidic pH due to the interaction between absorbent microparticles and ionizable micropollutants through electrostatic attraction or repulsion. The adsorption capacity increased with the decrease of solution pH and this phenomenon can be explained by the repulsive electrostatic interactions between the negative surface charge of LPGC and the negatively charged anionic form of DCF (-COO^−^) (Liang et al., 2019).

**Figure 9:**
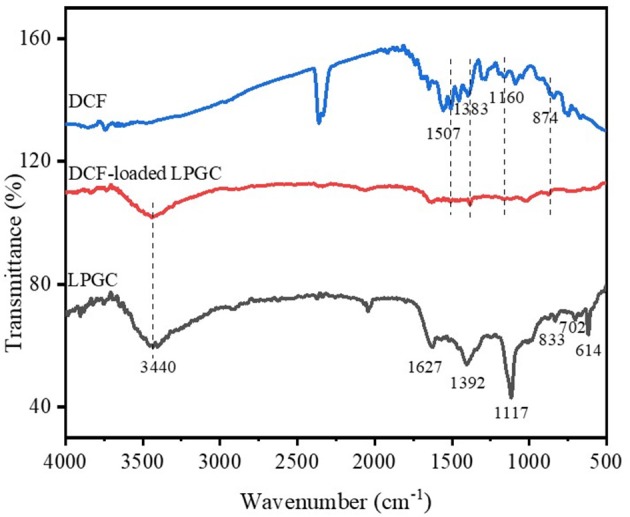
FT-IR spectra of LPGC, DCF and DCF-loaded LGBC.

**Figure 10:**
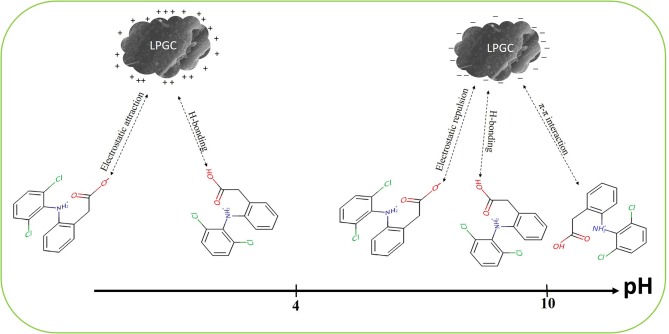
Possible mechanisms of DCF adsorption on to LPGC surface.

To investigate the possible adsorption mechanisms of the removal of DCF by LPGC, the FT-IR spectra of LPGC before and after DCF adsorption are shown in Figure 9 (scanning ranged from 500 to 4,000 cm^−1^). As can be seen from the FT-IR spectra, the left haft region above 2,000 cm^−1^ usually presents few common bands for all materials, while the right haft region shows a greater number of bands as well as their intensity variability. Among them, the broad peak at 3,440 cm^−1^ of original LPGC represented for the stretching vibration of O-H groups (dos Santos et al., 2019); and the peak located at 1,632 and 1,392 cm^−1^, were due to the skeletal vibration of aromatic C=C bonds (Jiang et al., 2015; Zhang et al., 2019). It can be seen that quite a few new peaks appeared at 1,507, 1,383, 1,160, and 874 cm^−1^, confirming the successful adsorbed DCF molecules on the surface of LPGC. Moreover, after DCF adsorption, the peak at 1,627 cm^−1^ shifted to 1,632 cm^−1^ and these peaks corresponded to the skeletal vibration of C=C bonds. This revealed the presence of a π-π interaction between aromatic rings of DCF and LPGC in the adsorption process (Wang et al., 2014). Moreover, Abo El Naga et al. (2019) reported that in relatively high pH conditions (pH ≥ 10), the adsorbent still had a considerable adsorption capacity, which might be attributed by the π-π electron donor acceptor interaction. Compared with the DCF adsorption capacity of LPGC at pH = 10, q_e_ reached a value of 86.3 mg g^−1^, which confirmed that the π-π interaction might be the mechanism for DCF removal by LPGC. It can be seen that the wavenumber peak at 1,117 cm^−1^ was prominent on LPGC, which attributed to C-O stretches (Guerrero et al., 2010), but it was no longer seen on DCF-loaded LPGC. It might shift to a small peak at 1,022 cm^−1^. A similar phenomenon appeared at the 614 cm^−1^ peaks and could be assigned for COO- stretching (Arivuselvi and Kumar, 2015). The intelligible changes indicated that the interaction between DCF microparticles and oxygen-containing functional groups of LPGC occurred. These functional groups might be H-bonding between the DCF and LPGC. Thus, it could be hypothesized that H-bonding interaction, along with electrostatic interaction and π-π interaction were the possible mechanisms of DCF adsorption by LPGC and the adsorption mechanism can is illustrated in Figure 10.

## Conclusion

In the present work, a porous biochar containing graphitic carbon (LPGC) was successfully synthesized from structurally separated pitch pine sawdust biomass using a two-step activation process. The H_2_SO_4_ treatment separated the biomass into a lignin component whereas K_2_FeO_4_ was adopted as an activated agent and catalyst for carbonization and graphitization processes, respectively. The one-step H_2_SO_4_-treated sample, K_2_FeO_4_-treated sample, and non-treated sample were used to investigate the advanced characteristics of as-proposed material. The obtained LPGC has a micro-mesoporous structure with a relatively high surface area (457.4 m^2^g^−1^), as well as the presence of a graphitic structure. Through these characteristics, LPGC showed an excellent adsorption capacity for DCF adsorption from an aqueous solution. Result from the Langmuir isotherm model indicated that the maximum adsorption capacity of LPGC for DCF was 159.7 mg g^−1^ at experimental conditions of temperature 298 K, contact time 24 h, and pH 6.5 and this is a considerable DCF adsorption capacity compared with other biomass-derived adsorbents. The kinetic and isotherm parameters were highly fitted with the Pseudo second-order kinetic model and Langmuir isotherms, respectively. The results of the thermodynamic study demonstrated that the DCF adsorption by LPGC process was spontaneous and endothermic. The adsorption capacity was significantly influenced by the pH solution, followed by an increasing trend with the decrease of pH value and obtained maximum adsorption capacity at the value of pH = 2.0. The adsorption process by LPGC was enhanced with the presence of NaCl in the DCF solution. In addition, the study about the effect of background electrolytes indicated that the adsorption capacity was positively affected by the presence of cations (Na^+^, K^+^, Ca^2+^, and Mg^2+^) but was negatively affected by the presence of anions (Cl^−^, NO${}_{3}^{-}$, SO${}_{4}^{2-}$, and PO${}_{4}^{3-}$). The possible adsorption mechanism that was expected, is that the H-bonding interaction, electrostatic interaction, and π-π interaction took part in the process of DCF removal from the solution by LPGC. Finally, from a sustainability perspective, the synthesis process of LPGC can be assessed as an advantageous method due to its ability to provide a cost-effective and efficient adsorbent for the removal of DCF from aqueous solutions.

## Data Availability Statement

The raw data supporting the conclusions of this article will be made available by the authors, without undue reservations, to any qualified researcher.

## Author Contributions

NT conducted the experimental work, wrote the first draft, and corrected the final manuscript. YL and SL supervised the work. HB, PZ, and MD further analyzed the data and worked on the subsequent version of the manuscript. XT and ML contributed to thorough discussions of the work.

## Conflict of Interest

The authors declare that the research was conducted in the absence of any commercial or financial relationships that could be construed as a potential conflict of interest.
