# Supplementary material for: Synthesis of Porous Biochar Containing Graphitic Carbon Derived From Lignin Content of Forestry Biomass and Its Application for the Removal of Diclofenac Sodium From Aqueous Solution
Source: Front Chem. 2020 Apr 23;8:274. doi: 10.3389/fchem.2020.00274 (PMC7212363; doi:10.3389/fchem.2020.00274)
Supplement: Supplementary file 1 [file Data_Sheet_1.pdf]

Supplementary Material

1 Model of data analysis

1.1 Kinetics study

Kinetic models provide direct information on the rate of diffusion or reaction (Tong et al., 2019). The adsorption kinetics of DCF onto LBC, C/LBC and BC was evaluated by the linearized pseudo first-order (1s) and pseudo second-order (2s) equations. The models are presented as follows:

$$ln\,(q_e - q_t) = lnq_e - k_1 t \qquad (1s)$$

$$\frac{t}{q_t} = \frac{1}{k_2 q_e^2} + \frac{1}{q_e} \qquad (2s)$$

Where q_e and q_t represent the adsorbed amount of DCF at equilibrium and at t time (mg/g), respectively while k_1 (1/min), k_2 (g/mg min) are rate constant of the pseudo-first-order model and pseudo-second-order model, respectively.

1.2 Isotherms study

Adsorption isotherm can describe the relation between the activity or equilibrium concentration of the adsorptive and the quantity of adsorbate on the surface at constant temperature. In current study, Langmuir and Freundlich models were used to fit adsorption data (Jiang et al., 2015). Langmuir isotherm model states that monolayer sorption occurs over an even adsorbent surface with no consequent interactions between adsorbed species (Abo El Naga et al., 2019) while Freundlich model can be used to describe multilayer adsorption occurring on an energetically heterogeneous adsorbent surface. The equilibrium state sorption data could be further applied for the non-linear fitting of following equations:

Langmuir isotherm equation:

$$q_e = \frac{K_L q_m C_e}{1+K_L C_e} \qquad (3s)$$

Where C_e is the concentration of DCF (mg/L) at equilibrium solution phase; q_e (mg/g) is the adsorption capacity at equilibrium point and q_m (mg/g) represents for the maximum adsorption amount corresponding to monolayer adsorption capacity of the adsorbent and K_L (L/mg) is the Langmuir isotherm constant.

Freundlich isotherm equation:

$$q_e = K_f C_e^{1/n} \qquad (4s)$$

Where C_e, q_e are similarly expressed with Eq. (3), n is the intensity of adsorption constant and K_f $[(mg/g)/(mg/L)^{1/n}]$ is the Freundlich adsorption affinity coefficient.

1.3 Thermodynamic study

The thermodynamic parameters including Gibbs free energy (ΔG^o), enthalpy (ΔH^o), and entropy (ΔS^o) of DCF on LBC are calculated by following equations (Jiang et al., 2015):

$$\Delta G^o = -RT\ln K_0 \quad (5s)$$

$$\Delta G^o = \Delta H^o - T\Delta S^o \quad (6s)$$

Where R (8.314 J/mol K) is universal gas constant and T (K) is the solution temperature in Kelvin. The thermodynamic equilibrium constant (K_0) is calculated by plotting $\ln K_d$ ($K_d = q_e/C_e$) versus C_e and extrapolating C_e to zero, while ΔS^o and ΔH^o can be obtained from the slope and intercept of ΔG^o versus T, respectively.

2 Supplementary Tables and Figures

2.1 Supplementary Tables

TABLE S1 Molecular structure and some physicochemical properties of diclofenac sodium (DrugBank, 2020)

Pharmaceutical trade name	Structure	Chemical formula	Molecular mass ($g\ mol^{-1}$)	Melting point (°C)	Water solubility ($mg\ mL^{-1}$)	pKa
Diclofenac sodium	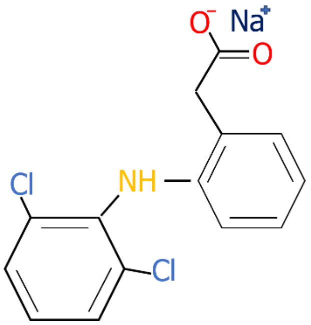	$C_{14}H_{10}Cl_2NNaO_2$	318.13	283-285	0.00482	4.15

2.2 Supplementary Figures

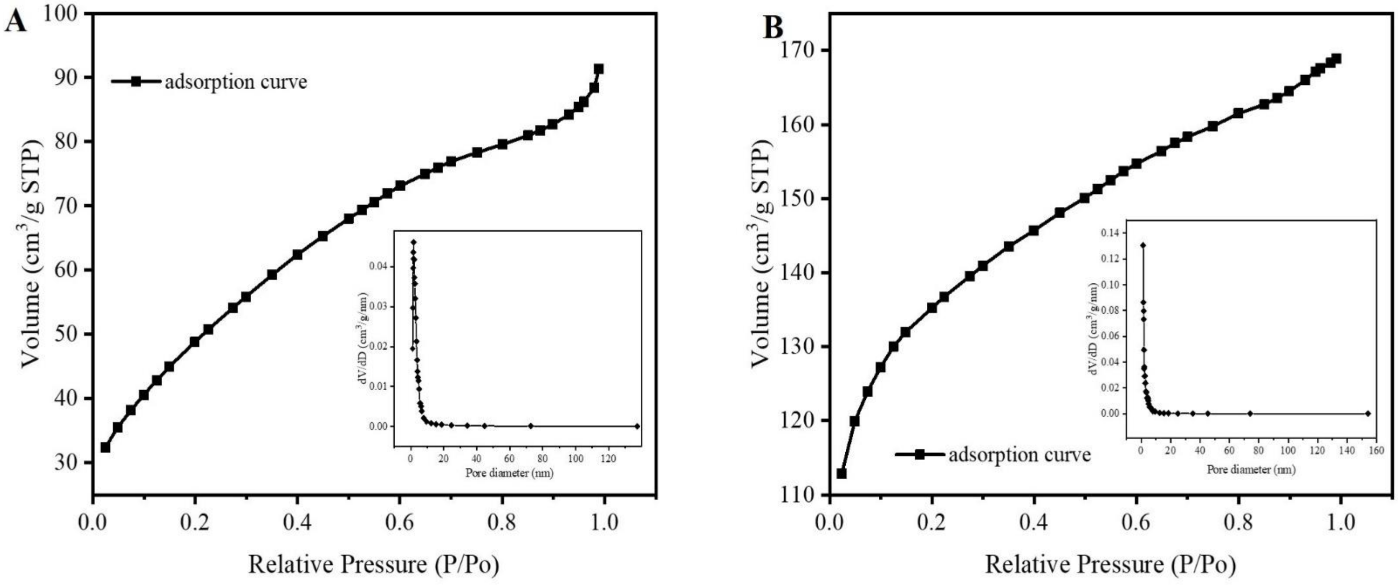

FIGURE S1 N_2 adsorption with the pores diameter distribution curves of **(A)** C/LBC and **(B)** BC

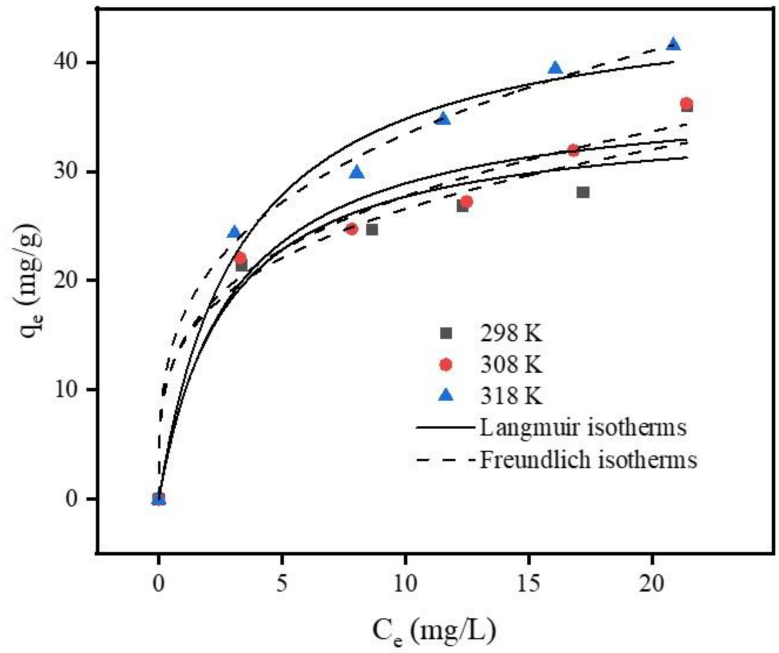

FIGURE S2 Adsorption isotherms of DCF by BC at three different temperatures

References

Abo El Naga, A.O., El Saied, M., Shaban, S.A., and El Kady, F.Y. (2019). Fast removal of diclofenac sodium from aqueous solution using sugar cane bagasse-derived activated carbon. *Journal of Molecular Liquids* 285**,** 9-19. doi: https://doi.org/10.1016/j.molliq.2019.04.062.

DrugBank (2020). Available: https://www.drugbank.ca/salts/DBSALT000466 [Accessed].

Jiang, L.-h., Liu, Y.-g., Zeng, G.-m., Xiao, F.-y., Hu, X., Hu, X., et al. (2015). Removal of 17β-estradiol by few-layered graphene oxide nanosheets from aqueous solutions: External influence and adsorption mechanism. *Chemical Engineering Journal* 284. doi: 10.1016/j.cej.2015.08.139.

Tong, Y., McNamara, P., and Mayer, B. (2019). Adsorption of organic micropollutants onto biochar: A review of relevant kinetics, mechanisms and equilibrium. *Environmental Science: Water Research & Technology* 5. doi: 10.1039/C8EW00938D.
